# Matrix-assisted laser desorption/ionization time of flight mass spectrometry for comprehensive indexing of East African ixodid tick species

**DOI:** 10.1186/s13071-016-1424-6

**Published:** 2016-03-15

**Authors:** Julian Rothen, Naftaly Githaka, Esther G. Kanduma, Cassandra Olds, Valentin Pflüger, Stephen Mwaura, Richard P. Bishop, Claudia Daubenberger

**Affiliations:** International Livestock Research Institute (ILRI), PO Box 30709-00100, Nairobi, Kenya; Department of Medical Parasitology and Infection Biology, Clinical Immunology Unit, Swiss Tropical and Public Health Institute (Swiss TPH), Socinstr. 57, CH 4002 Basel, Switzerland; University of Basel, Petersplatz 1, CH 4003 Basel, Switzerland; Mabritec SA, Lörracherstrasse 50, CH 4125 Riehen, Switzerland; Department of Veterinary Microbiology and Pathology, Washington State University, PO Box 647040, Pullman, WA 99163 USA; Biosciences eastern and central Africa – International Livestock Research Institute (BecA-ILRI) Hub, PO Box 30709, 00100 Nairobi, Kenya; Department of Biochemistry, School of Medicine, University of Nairobi, PO Box 30197, Nairobi, Kenya

**Keywords:** MALDI-TOF MS, Ticks, Species identification, Vector epidemiology, COI, *Amblyomma*, *Boophilus*, *Hyalomma*, *Rhipicephalus*

## Abstract

**Background:**

The tick population of Africa includes several important genera belonging to the family Ixodidae. Many of these ticks are vectors of protozoan and rickettsial pathogens including *Theileria parva* that causes East Coast fever, a debilitating cattle disease endemic to eastern, central and southern Africa. Effective surveillance of tick-borne pathogens depends on accurate identification and mapping of their tick vectors. A simple and reproducible technique for rapid and reliable differentiation of large numbers of closely related field-collected ticks, which are often difficult and tedious to discriminate purely by morphology, will be an essential component of this strategy. Matrix-assisted laser desorption/ionization time of flight mass spectrometry (MALDI-TOF MS) is increasingly becoming a useful tool in arthropod identification and has the potential to overcome the limitations of classical morphology-based species identification. In this study, we applied MALDI-TOF MS to a collection of laboratory and field ticks found in Eastern Africa. The objective was to determine the utility of this proteomic tool for reliable species identification of closely related afrotropical ticks.

**Methods:**

A total of 398 ixodid ticks from laboratory maintained colonies, extracted from the hides of animals or systematically collected from vegetation in Kenya, Sudan and Zimbabwe were analyzed in the present investigation. The cytochrome *c* oxidase I (COI) genes from 33 specimens were sequenced to confirm the tentatively assigned specimen taxa identity on the basis of morphological analyses. Subsequently, the legs of ticks were homogenized and analyzed by MALDI-TOF MS. A collection of reference mass spectra, based on the mass profiles of four individual ticks per species, was developed and deposited in the spectral database SARAMIS™. The ability of these superspectra (SSp.) to identify and reliably validate a set of ticks was demonstrated using the remaining individual 333 ticks.

**Results:**

Ultimately, ten different tick species within the genera *Amblyomma*, *Hyalomma*, *Rhipicephalus* and *Rhipicephalus (Boophilus)* based on molecular COI typing and morphology were included into the study analysis. The robustness of the 12 distinct SSp. developed here proved to be very high, with 319 out of 333 ticks used for validation identified correctly at species level. Moreover, these novel SSp. allowed for diagnostic specificity of 99.7 %. The failure of species identification for 14 ticks was directly linked to low quality mass spectra, most likely due to poor specimen quality that was received in the laboratory before sample preparation.

**Conclusions:**

Our results are consistent with earlier studies demonstrating the potential of MALDI-TOF MS as a reliable tool for differentiating ticks originating from the field, especially females that are difficult to identify after blood feeding. This work provides further evidence of the utility of MALDI-TOF MS to identify morphologically and genetically highly similar tick species and indicates the potential of this tool for large-scale monitoring of tick populations, species distributions and host preferences.

**Electronic supplementary material:**

The online version of this article (doi:10.1186/s13071-016-1424-6) contains supplementary material, which is available to authorized users.

## Background

As obligate hematophagous organisms, ticks can acquire and transmit pathogenic microorganisms such as eukaryotic parasites, bacteria, viruses and fungi both through vertical transmission or when feeding on their hosts [1]. Tick-borne diseases (TBDs) cause significant economic losses to the cattle industry in tropical and subtropical regions of the world [2]. Some tick species are capable of building up focally highly dense populations, causing additional production losses in farm animals from irritation, skin damage and accompanying chronic inflammation, blood loss and in some cases, secondary infections [1, 3]. In most of Eastern Africa, several ixodid tick species share overlapping habitats and multiple tick infestations in livestock is frequently observed [4]. The control of TBDs can be improved and targeted appropriately by accurate monitoring of tick vectors. This has traditionally been done by examining morphological features using a light microscope, and with the aid of taxonomical descriptions and illustrations [4, 5]. Unfortunately, the expert knowledge required for this task is rare in most settings where TBDs are endemic [4]. In addition, damaged or immature tick stages, or replete female ticks are often difficult to identify accurately based on morphological features alone [6]. Molecular approaches like sequencing of the cytochrome *c* oxidase subunit I (COI), the 12S rDNA or the internal transcribed spacer 2 (ITS2) can overcome the limitations of conventional tick taxonomy. Due to the labor, time and costs involved in DNA extraction, PCR amplification, purification and nucleotide sequencing, this approach is typically limited to well-equipped laboratories [7, 8]. When COI, 12S or ITS reference sequences are scarce or missing from public nucleotide databases, it is difficult to conclusively resolve the species level thus non-identical sequences may remain unidentifiable [9]. Additionally, public databases are known to sometimes contain misidentified species, and sequences showing errors or obtained from contaminated samples resulting in inaccurate classification [10]. Hence, a marker-based identification system is useful to supplement morphological species identification and support taxonomy, either as corroborating evidence for existing hypotheses or as a starting point for further testing using additional techniques [11].

Matrix assisted laser desorption/ionization time of flight mass spectrometry (MALDI-TOF MS) is emerging as an alternative to both morphology and PCR-based typing for identifying disease vectors such as mosquitoes [12, 13], tsetse fly [14] and ticks [15, 16]. MALDI-TOF MS makes use of a small quantity of whole organism material, and thus can identify damaged tick specimens or immature stages [16]. As a diagnostic technique, MALDI-TOF MS is both cost-effective and rapid, can be performed without in depth technical knowledge and the data results have been found to be highly reproducible [17]. In disease endemic areas, MALDI-TOF MS could assist in resolving questions that are difficult to answer with traditional morphological or current molecular based typing methods. For example, the unclear epidemiological status of the two closely related tick species *Rhipicephalus simus* and *Rhipicephalus praetextatus* in Kenya, where COI sequencing of field samples strongly indicates occurrence of *R. simus*, although this species is currently thought to be confined to Southern Africa [4]. It is possible that ixodid species other than *Rhipicephalus appendiculatus* may transmit *Theileria parva* in Eastern Africa, because number of *Rhipicephalus* species on domestic animals is greater than previously thought (E. Kanduma and R. Bishop, unpublished data), but more in-depth, higher resolution analyses of both tick vectors and the pathogens they transmit are necessary to confirm this. Moreover, the vector of *Theileria* sp. (buffalo) a species that is infective to cattle at livestock-wildlife interface with unknown consequences in respect of pathology, especially in the co-infection situation is currently unknown [18]. These and similar questions require resolution especially in the context of the epidemiology of theileriosis at the livestock-wildlife interface [19, 20]. Methods endowed with higher resolution and throughput ideally for both the tick vectors and the pathogens that they transmit will be required in future to follow tick borne disease epidemiology, particularly in times of rapid climate changes in these regions [18]. The objective of this study was to extend the application of MALDI-TOF as a high-throughput tick typing method [15, 16] to a collection of Afrotropical ixodid ticks obtained from Eastern Africa. We envisage that the data from our tick collection will serve as a reference for indexing the multiple ixodid tick species that frequently occur sympatrically in Africa.

## Methods

### Laboratory reared ticks

A total of 398 adult ticks built the basis of this study. One hundred fifty six ticks were obtained from colonies that had been bred and maintained as closed genetic stocks at the International Livestock Research Institute (ILRI) Tick Unit. These were reared and managed as described by Bailey [21] and Irvin and Brocklesby [22]. With the exception of a *Hyalomma* sp. whose identity was uncertain until recently, the history and identity of all other tick species kept at the unit were well documented (Table 1). The laboratory maintained colonies ticks consisted of *Ambylomma variegatum* (21), *Hyalomma* sp. (16)*, R. appendiculatus* (40 (Muguga colony) and 9 (Kiambu colony)), *Rhipicephalus* (*Boophilus*) *decoloratus* (22), *Rhipicephalus* (*Boophilus*) *microplus* (21) and *Rhipicephalus evertsi evertsi* (27). Ticks were transferred to 70 % ethanol and shipped at room temperature to Basel for the MALDI-TOF MS analysis and genomic DNA extraction.

**Table 1:** Overview of 398 ticks that built the basis of this study

Morphologically assigned species name	Quantity	Sex	Geographical origin	Source
*Amblyomma gemma*	21	4 F, 15 M, 2 ND	Kenya	Vegetation & Animal
*Amblyomma hebraeum*	4	2 F, 1 M, 1 ND	Zimbabwe	Vegetation
*Amblyomma variegatum*	21	4 F, 15 M, 2 ND	Kenya	Lab colony
*Amblyomma variegatum*	19	5 F, 14 M	Kenya	Vegetation & Animal
*Hyalomma anatolicum anatolicum*	13	10 F, 1 M, 2 ND	Sudan	Vegetation
*Hyalomma dromedarii*	3	2 M, 1 ND	Kenya	Vegetation & Animal
*Hyalomma marginatum rufipes*	18	5 F, 11 M, 2 ND	Kenya	Vegetation & Animal
*Hyalomma truncatum*	14	5 F, 6 M, 3 ND	Kenya	Vegetation & Animal
*Hyalomma* sp.	16	8 F, 6 M, 2 ND	Kenya	Lab colony
*Rhipicephalus (Boophilus) decoloratus*	22	21 F, 1 M	Kenya	Lab colony
*Rhipicephalus (Boophilus) decoloratus*	19	19 F	Kenya & Sudan	Animal
*Rhipicephalus (Boophilus) microplus*	21	21 F	Kenya	Lab colony
*Rhipicephalus (Boophilus) microplus*	3	3 F	Kenya	Animal
*Rhipicephalus appendiculatus*	40	16 F, 23 M, 1 ND	Kenya	Lab colony (Muguga)
*Rhipicephalus appendiculatus*	38	15 F, 22 M, 1 ND	Kenya	Vegetation & Animal
*Rhipicephalus appendiculatus*	9	8 F, 1 ND	Kenya	Lab colony (Kiambu)
*Rhipicephalus evertsi evertsi*	27	10 F, 16 M, 1 ND	Kenya	Lab colony
*Rhipicephalus evertsi evertsi*	28	7 F, 20 M, 1 ND	Kenya	Vegetation & Animal
*Rhipicephalus praetextatus*	8	5 F, 2 M, 1 ND	Kenya	Vegetation & Animal
*Rhipicephalus pulchellus*	37	21 F, 14 M, 2 ND	Kenya	Vegetation & Animal
*Rhipicephalus simus*	17	8 F, 7 M, 2 ND	Kenya	Vegetation & Animal
Total	398			

*ND* sex not determined, *M* male, *F* female

### Field ticks and identification by morphological characteristics

**Fig. 1:**
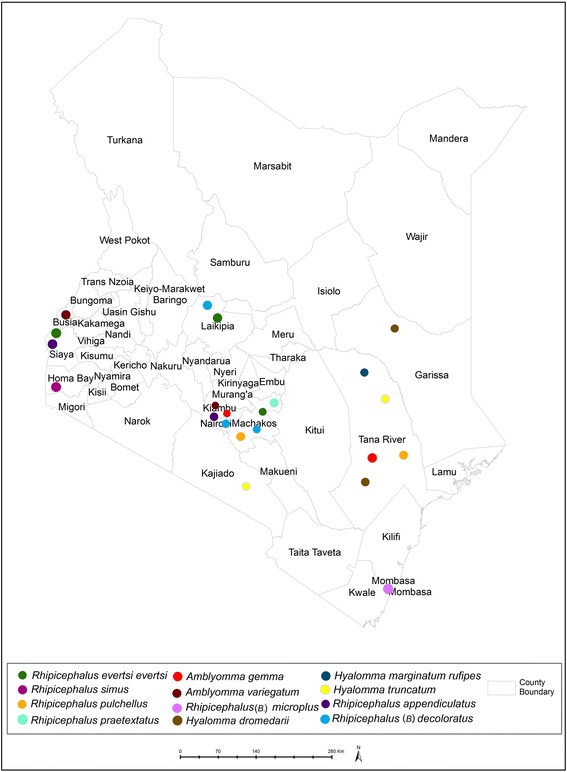
Geographical origin of ticks used for MALDI-TOF MS analysis in Kenya. The collection sites of the ambiguous *Hyalomma* species and the specimens obtained from outside Kenya are not shown

Two hundred forty two field ticks were either plucked directly from animal hosts (cattle/sheep) or were collected from pastures/vegetation. One hundred sixty of these ticks were collected 2014 at various sites in Kenya (Fig. 1). The remaining 82 specimens were collected from multiple localities within East Africa during a study investigating the population structure of *R. appendiculatus* in the field [23] (Fig. 1). Ticks were assigned to sex and species as described by Hoogstraal (1956) and Walker (2000 and 2003) [4, 5] and stored in 70 % ethanol and kept at 4 °C prior to shipping to Basel. Due to the high proportion (242/398) of field ticks and the inclusion of both male and female specimens, we expected our collection to reliably reflect intraspecies physiological and molecular diversity.

### Molecular COI gene typing

#### DNA extraction & PCR

To confirm the morphologically assigned species identities of field ticks and to check for potential molecular differences to laboratory ticks, specimens of both origins were subjected to molecular analysis. A tick was randomly chosen from the library and thoroughly rinsed with distilled water in order to remove any ethanol residues. The tick legs were detached with a scalpel and stored in 70 % ethanol for later MALDI-TOF MS analysis. The tick body was transferred to a 1.5 ml micro-centrifuge tube and placed in liquid nitrogen for 5 min. Using a polypropylene pestle (Sigma-Aldrich), the frozen tick body was thoroughly grinded to powder. Whole genomic DNA was extracted using the QIAGEN® *DNeasy*® *Blood & Tissue Kit* (Qiagen GmbH, Germany). One hundred eighty microliter buffer ATL and 20 μl proteinase K were added to the grinded tick body and the mixture incubated overnight at 56 °C to ensure complete lysis of the tissue. The further extraction steps were carried out according to the manufacturers’ protocol. The final concentration of extracted gDNA was determined with a spectrophotometer (WPA Lightwave II, Biochrom). Cytochrome *c* oxidase subunit I (COI) gene sequences of individual ticks were obtained by PCR amplification using the forward primer LCO149021 (5′-GGTCAACAAATCATAAAGATATTGG-3′) and reverse primer HC02198 (5′ TAAACTTCAGGGTGACCAAAAAATCA-3′) [24]. PCR was performed in a 50 μl reaction consisting of 5 μl 10× PCR Buffer (containing 10 mM MgCl_2_), 1 μl dNTP mix, 1 μl MgCl_2_, 2.5U HotStarTaq DNA Polymerase (Qiagen GmbH, Germany), 1 μl each of forward and reverse primers and 25 to 500 ng of tick gDNA as a template. The final volume of the reaction mixture was made up to 50 μl with nuclease-free water (Thermo Scientific, Germany). The PCR conditions consisted of an initial heat activation step at 95 °C for 15 min followed by 35 cycles of denaturation at 94 °C for 1 min, annealing at 45 °C for 1 min and extension at 72 °C for 1 min. The final extension was performed for 10 min at 72 °C. Per run, between 5 to 15 reactions were amplified, including two reactions where the gDNA template was omitted and compensated with nuclease-free water, serving as negative controls. The quality of the PCR products was determined by running 5 μl of stained (DNA-Dye NonTox, PanReac/AppliChem) DNA on a 1.0 % agarose gel. The bands were visualized and examined with the GelDoc™ EZ Imager (BioRAD). The amplified COI products were purified using the QIAquick PCR Purification Kit (Qiagen GmbH, Germany) following the manufacturers’ protocol. DNA samples were eluted with 40 μl elution buffer (10 mM TrisCl). The COI gene was sequenced using the gene specific forward and reverse primer pair used for PCR amplification at Microsynth AG, Switzerland.

#### Data analysis: sequence editing and multiple alignments

COI sequence chromatograms were visually inspected and manually edited using Seqtrace [25]. Using the Molecular Evolutionary Genetic Analysis (MEGA) software version 6.0 [26], consensus sequences were generated from a forward and reverse sequence for each of the COI PCR products. Species identity was confirmed by matching of the consensus sequences with reference data deposited in the NCBI GenBank [27] and/or the BOLD database [28], a barcoding database that is a component of the Tree of Life project and contains only COI nucleotide sequences. A positive match with a GenBank record was defined as more than 95 % query coverage and ≥ 97 % identity. A positive match with a record on BOLD was declared at identity values ≥ 97 %. Multiple sequence alignment analysis of all consensus sequences was performed using the MUSCLE tool in MEGA. The nucleotide sequences were trimmed to around 680 bp and the phylogenetic analyses computed based on maximum likelihood algorithm and the tree file exported to FigTree [29] for final editing.

### MALDI-TOF MS analysis of ticks

#### Sample preparation

The sample processing protocol has been adopted from previous studies [15, 30] and modified accordingly. Specimens were removed from the library, rinsed once with distilled water and dried on absorbent paper. Depending on the size of the tick, two to eight legs were detached with a scalpel and placed in a 1.5 ml microcentrifuge tube containing 10 μl of 25 % formic acid. The samples were homogenized using a stainless steel micropestle (LLG Labware, Switzerland) powered by a portable drilling machine for 30 s. The homogenate was then centrifuged at 10,000 rpm for 1 min and 1 μl of the supernatant transferred into a microcentrifuge tube containing 8 μl of matrix solution (saturated sinapinic acid, 60 % acetonitrile, 40 % high-performance liquid chromatography (HPLC)-grade water and 0.3 % trifluoroacetic acid). After thoroughly mixing, the solution was spotted in quadruplicates (1 μl each) on a steel target plate (Mabritec AG, Basel, Switzerland). The spots were allowed to dry for several minutes until crystallization of the matrix/analyte mixture was complete and the target plate thereafter transferred to the MALDI-TOF MS instrument.

#### MALDI-TOF parameters

The MS measurements were carried out using a MALDI-TOF Mass Spectrometry Axima™ Confidence machine (Shimadzu-Biotech Corp., Kyoto, Japan) with detection in the linear positive mode, allowing the interrogation of high molecular weight samples. The acceleration voltage was set by default to 20 kV with an extraction delay time of 200 ns and a laser frequency of 50 Hz. The analysis was carried out in the mass range between 4000 and 20,000 Da. To ensure an even measurement covering the entire area of the sample spot, a netlike pattern of 100 equally distributed locations was defined. At 50 of these profiles, 10 consecutive laser shots were applied, adding up to 500 laser shots per sample spot. The ion gate was set at 3900 Da and the pulsed extraction optimized at 12,000 Da. The generated raw spectra were processed with the Launchpad™ version 2.9 software (Shimadzu-Biotech Corp., Kyoto, Japan) using the following settings: the advanced scenario was chosen from the parent peak cleanup menu, peak width was set to 80 channels, smoothing filter width to 50 channels, baseline filter width to 500 channels and the threshold apex was chosen as the peak detection method. The threshold apex peak detection was set as a dynamic type and the offset was set to 0.020 mV with a response factor of 1.2. The processed spectra were exported as peak lists with m/z values for each peak and signal intensity in the ASCII format. Each target plate was externally calibrated using the reference spectra of *Escherichia coli* strain DH5α.

#### Spectral analysis: superspectrum design & validation

The generated mass spectra were exported to Launchpad™, quality-checked by eye and repeat measurements carried out if necessary. Mass spectra of reviewed spectra were then transferred in ASCII format to the spectral archive and microbial identification system (SARAMIS™) (AnagnosTec, Potsdam-Golm, Germany). A biomarker mass pattern, called superspectrum (SSp.) was calculated for each tick species using the SARAMIS™ SuperSpectra™ tool. To that end, the quadruplicate mass lists of four ticks were consolidated, peaks with a relative intensity below 1 % removed and average masses calculated with an error of 800 ppm. Masses of high species specificity were determined by comparison between the different tick SSp. and weighted manually.

In the validation step, using the SARAMIS™ identification tool, quadruplicate mass spectra of the remaining ticks were matched against the previously designed reference superspectra. A match between a SSp. and acquired mass spectra was regarded as positive at 75 % identity or higher. Accordingly, each mass spectrum achieved either a single match, sharing ≥75 % identity with only one SSp., a multiple match if sharing ≥ 75 % identity with more than one SSp., or no match if the 75 % identity threshold with no SSp. was reached. In case of a multiple match, the SSp. achieving the highest identity score was assumed the valid match. Subsequently, a given tick was assigned a final ID (i.e. species identification) when two criteria were met: (1) at least one of the four mass spectra matched to a SSp. and (2) assigned matches amongst the four mass spectra were not in conflict with each other.

### Ethical statement

ILRI’s Institutional Animal Care and Use Committee (IACUC) governed the use of cattle and rabbits for the maintenance of the lab tick colonies (approval no. 2010.1). The collection of field ticks did not involve endangered or protected species.

## Results

### Morphological identification of field ticks

Morphological identification grouped the ticks collected from vegetation and animals into 14 different species (Table 1). While five of these species were already represented by the laboratory colonies, nine species were exclusively covered by field ticks only. These species included *Amblyomma gemma* (21), *Amblyomma hebraeum* (4), *Hyalomma anatolicum anatolicum* (13), *Hyalomma dromedarii* (3), *Hyalomma marginatum rufipes* (18), *Hyalomma truncatum* (14), *R. praetextatus* (8), *Rhipicephalus pulchellus* (37) and *R. simus* (17).

Laboratory reared and field ticks combined, the 398 ticks grouped into 14 species within three genera, namely *Amblyomma* (3), *Hyalomma* (4) and *Rhipicephalus* (5)/*Rhipicephalus (Boophilus)* (2). 51.3 % (204/398) of the ticks belonged to the genus *Rhipicephalus*, 16.3 % (65/398) to *Rhipicephalus* (*Boophilus*), 16.3 % (65/398) to *Amblyomma* and 16.1 % (64/398) to *Hyalomma*. Six tick species including *R. appendiculatus* (87), *R. evertsi evertsi* (55), *R.* (*B.*) *decoloratus* (41), *A. variegatum* (40) and *R. pulchellus* (37) represented 65.3 % of the collection (Table 1).

### COI gene sequencing

For a total of 33 ticks, COI gene sequences were obtained (Table 2). No unspecific amplification occurred for the negative controls included in each PCR amplification run. The sequenced amplicons - with the exception of three ticks where no consensus sequence could be determined - were all approximately 700 bp in size, and the sequences were used for comparison against entries at GenBank and/or BOLD. Since there are no COI gene sequences for *A. gemma* currently deposited in GenBank, specimen identity for these ticks was assigned solely based on the BOLD entries. Two ticks morphologically identified as *A. hebraeum* matched clearly with reference records of *A. gemma* in the BOLD database (identity scores of 100 and 99.70 %). At the same time, the identity shared with NCBI reference sequences for *A. hebraeum* was only 89 %. The whole group of four specimens morphologically determined as *A. hebraeum*, were henceforth assigned as *A. gemma*. The morphologically unidentified *Hyalomma* sp. was clearly determined as *H. dromedarii*, with four COI sequenced ticks matching with high scores to the respective reference records in both databases. The species designation was adopted accordingly for the further course of this study. Two members of the morphologically identified *R. simus* ticks both matched with the *R. simus* reference sequence (AF132840.1) present in NCBI with a score of 92 % (data not shown) and with a slightly higher score (94.5 %) to *R. praetextatus* in the BOLD database. Higher molecular identity (99 % with only 67 % query cover) was achieved with deposited partial COI gene sequences (472 bp) of *Rhipicephalus muhsamae*. The two ticks morphologically identified as *R. praetextatus* matched with a relatively high identity of 96 % (data not shown) to a *R. simus* record on NCBI and with 100 % identity to unpublished *R. praetextatus* records on BOLD. Given these uncertain molecular results and the limited reference records available, the specimens of the *R. simus* and *R. praetextatus* batch were merged to one group and the species designation changed to *R. simus* group. Extraction of DNA qualitatively sufficient for COI gene sequencing failed with all specimens of *H. anatolicum anatolicum*. The morphologically assigned species identity of these ticks could therefore not be confirmed on molecular basis. The COI gene sequence of a tick (ID 139, Table 2) morphologically identified as *H. marginatum rufipes* was identified with equal score as *H. marginatum rufipes* and *H. truncatum* on GenBank. Since the same COI sequence matched highest to *H. marginatum rufipes* record on the BOLD database, the species identity as initially determined on a morphological basis was assumed correct. Taking together the molecular results, all ticks morphologically identified as *A. hebraeum, Hyalomma* sp., *R. simus* and *R. praetextatus* were reclassified to *A. gemma*, *H. dromedarii* and *R. simus* group*,* respectively. The species identity of *H. anatolicum anatolicum* could not be confirmed due to insufficient quality of genomic DNA. All the remaining tick species that were assigned morphologically were confirmed by our molecular analysis.

**Table 2:** Tabular overview of 33 ticks additionally identified by COI molecular typing

			Morphological identification	BOLD identification	GenBank identification	
Tick ID	COI gene length [bp]	Origin	Species ID	Species ID (Identity)	Species ID (Accession Nr.)	Identity
**154**	711	Field	*A. gemma*	*A. gemma* (99.80 %)	*no reliabe ID*	
**183**	688	Field	*A. gemma*	*A. gemma* (100 %)	*no reliabe ID*	
32	687	Field	*A. hebraeum*	*A. gemma* (100 %)	*no reliabe ID*	
109	686	Field	*A. hebraeum*	*A. gemma* (99.70 %)	*no reliabe ID*	
**36**	692	Lab	*A. variegatum*	*A. variegatum* (100 %)	*A. variegatum* (GU062743.1)	97 %
86	651^a^	Field	*A. variegatum*	*A. variegatum* (99.70 %)	*A. variegatum* (GU062743.1)	99 %
**242**	702	Lab	*A. variegatum*	*A. variegatum* (100 %)	*A. variegatum* (GU062743.1)	97 %
27	688	Field	*H. dromedarii*	*H. dromedarii* (100 %)	*H. dromedarii* (AJ437071.1)	99 %
74	688	Field	*H. dromedarii*	*H. dromedarii* (100 %)	*H. dromedarii* (AJ437061.1)	99 %
118	680	Field	*H. dromedarii*	*H. dromedarii* (100 %)	*H. dromedarii* (AJ437071.1)	99 %
**34**	688	Lab	*H. sp.*	*H. dromedarii* (100 %)	*H. dromedarii* (AJ437061.1)	99 %
**112**	686	Lab	*H. sp.*	*H. dromedarii* (100 %)	*H. dromedarii* (AJ437061.1)	99 %
**194**	680	Lab	*H. sp.*	*H. dromedarii* (100 %)	*H. dromedarii* (AJ437061.1)	99 %
**207**	686	Lab	*H. sp.*	*H. dromedarii* (100 %)	*H. dromedarii* (AJ437061.1)	99 %
**139**	689	Field	*H. m. rufipes*	*H. m. rufipes* (99.84 %)	*H. m. rufipes* (AJ437100.1)	99 %
					*H. truncatum* (AJ437088.1)	99 %
**359**	688	Field	*H. m. rufipes*	*H. m. rufipes* (99.12 %)	*H. m. rufipes* (AJ437095.1)	99 %
**72**	684	Field	*H. truncatum*	*H. truncatum* (99 %)	*H. truncatum* (AJ437084.1)	97 %
**361**	555^a^	Field	*H. truncatum*	*H. truncatum* (98 %)	*H. truncatum* (AJ437084.1)	97 %
**38**	693	Lab (Kiambu)	*R. appendiculatus*	*R. appendiculatus* (99.50 %)	*R. appendiculatus* (AF132833.1)	97 %
**121**	687	Lab (Muguga)	*R. appendiculatus*	*R. appendiculatus* (99.50 %)	*R. appendiculatus* (AF132833.1)	98 %
**198**	679	Lab (Muguga)	*R. appendiculatus*	*R. appendiculatus* (99.50 %)	*R. appendiculatus* (AF132833.1)	98 %
**225**	687	Field	*R. appendiculatus*	*R. appendiculatus* (99.85 %)	*R. appendiculatus* (AF132833.1)	99 %
146	673	Field	*R. (B.) decoloratus*	*R. (B.) decoloratus* (100 %)	*R. (B.) decoloratus* (AF132826.1)	99 %
**278**	690	Lab	*R. (B.) decoloratus*	*R. (B.) decoloratus* (99.85 %)	*R. (B.) decoloratus* (AF132826.1)	99 %
**165**	585^a^	Lab	*R. (B.) microplus*	*R. (B.) microplus* (100 %)	*R. (B.) microplus* (KC503261.1)	100 %
**377**	689	Field	*R. (B.) microplus*	*R. (B.) microplus* (100 %)	*R. (B.) microplus* (KC503261.1)	99 %
**170**	694	Field	*R. evertsi evertsi*	*R. evertsi evertsi* (100 %)	*R. evertsi evertsi* (AF132835.1)	98 %
**275**	688	Lab	*R. evertsi evertsi*	*R. evertsi evertsi* (100 %)	*R. evertsi evertsi* (AF132835.1)	98 %
370	688	Field	*R. praetextatus*	*no reliabe ID*	*no reliabe ID*	
396	702	Field	*R. praetextatus*	*no reliabe ID*	*no reliabe ID*	
**397**	702	Field	*R. pulchellus*	*R. pulchellus* (98 %)	*R. pulchellus* (AY008682.1)	99 %
**29**	701	Field	*R. simus*	*no reliabe ID*	*no reliabe ID*	
**115**	690	Field	*R. simus*	*no reliabe ID*	*no reliabe ID*	

^a^no consensus sequence

Marked in bold: Ticks later used for SSp. design

No reliable ID: Identity with top match < 97 %

### MALDI-TOF MS analysis

#### Spectra quality

A total of 1592 single mass spectra were generated, corresponding to 398 ticks measured in quadruplicate. Inadequate spectral quality was assessed visually and accordingly 55 ticks were re-measured and integrated into the sample set. Seventeen ticks were excluded from the study after the first MALDI-TOF MS measurement since the poor overall state of the specimens did not allow for the generation of qualitatively adequate mass spectra. Among the excluded ticks were four ticks of the *R. simus* group batch that were partially overgrown by fungus. The entire collection (13 specimens) of *H. anatolicum anatolicum* for which also preceding DNA extraction had failed, were stored in leaky microtubes and as a result completely desiccated. The remaining 1524 mass spectra (381 ticks) used for the subsequent analyses presented a good signal-to-noise ratio and clear protein peaks, mostly distributed between 4000 to 13,000 Da (Additional file 1: Figure S1). The number of protein peaks per spectrum ranged from 14 to 145, with an average peak count of 42.5.

#### Intra species reproducibility of mass spectra

**Fig. 2:**
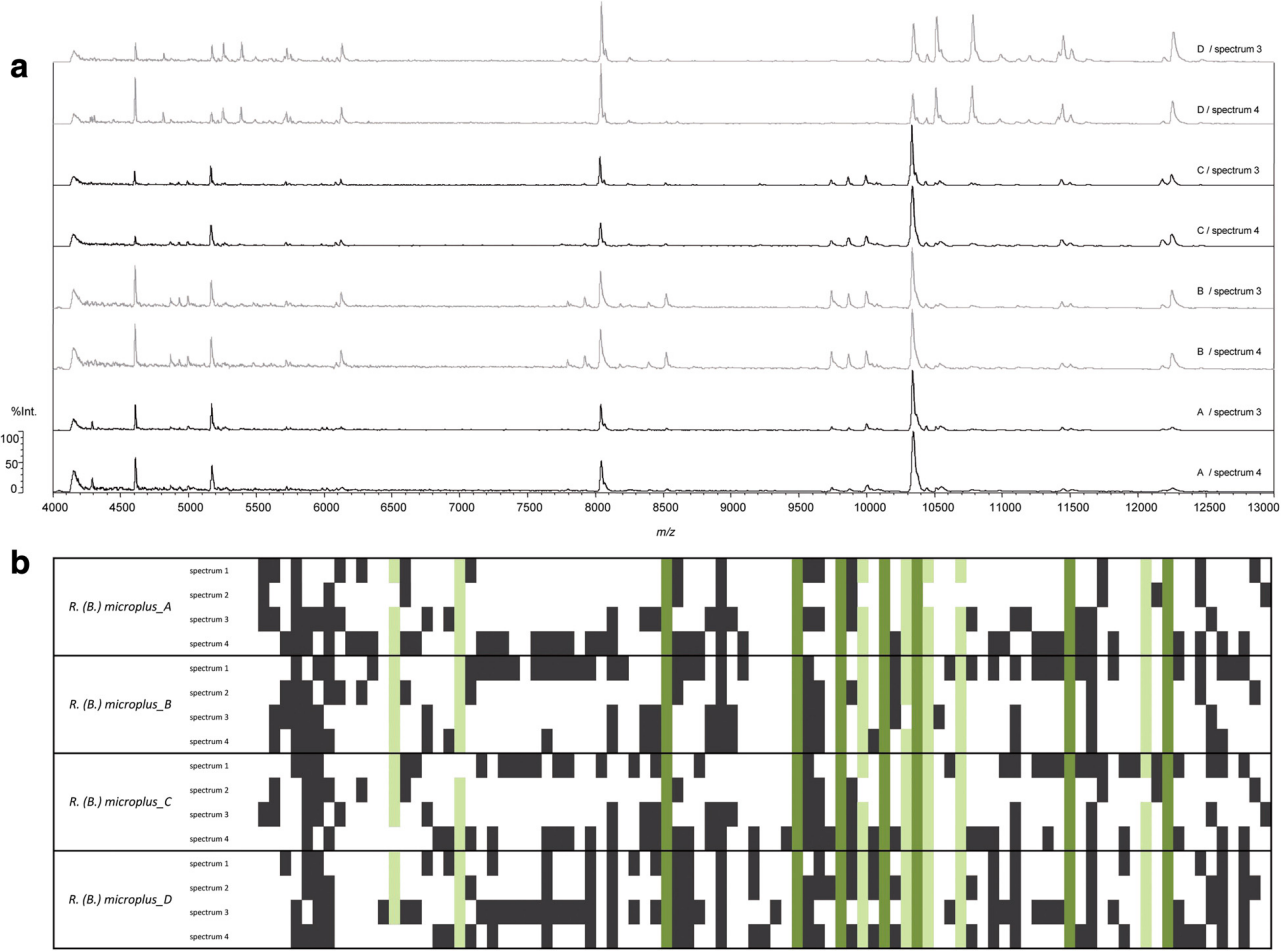
Comparison of *R.* (*B*) *microplus* mass profiles to assess intraspecies reproducibility. **a** Visual comparison of spectral profiles derived from four specimens (A–D) indicates both significant similarity as well as individual differences in mass fingerprints. **b** Comparative analysis of 16 mass spectra, corresponding to ticks A–D measured in quadruplicates (spectrum 1–4). Vertically arranged are the total 93 different masses found amongst the 16 spectra. A *white field* indicates absence, a *black* or *green field* presence of the given mass in the respective spectrum. Highly conserved protein masses are marked in *dark green* (100 % abundance) or *light green* (abundant in at least 3 out of 4 technical replicates in all specimens)

Visual inspection of spectral profiles revealed a generally highly similar mass fingerprint shared between individuals of the same species. Simultaneously, mass spectra were still heterogeneous on species level with missing or exclusive mass peaks only present in certain specimens or spectral profiles (Fig. 2). However, comparative analysis of the spectral profiles with SARAMIS™ confirmed that few major protein peaks remained highly conserved within a given species (Fig. 2). A significant difference between the mass spectra of male and female ticks of the same species could not be observed. This is in line with what has been reported in other studies [31, 32].

#### Inter species specificity of mass spectra

**Fig. 3:**
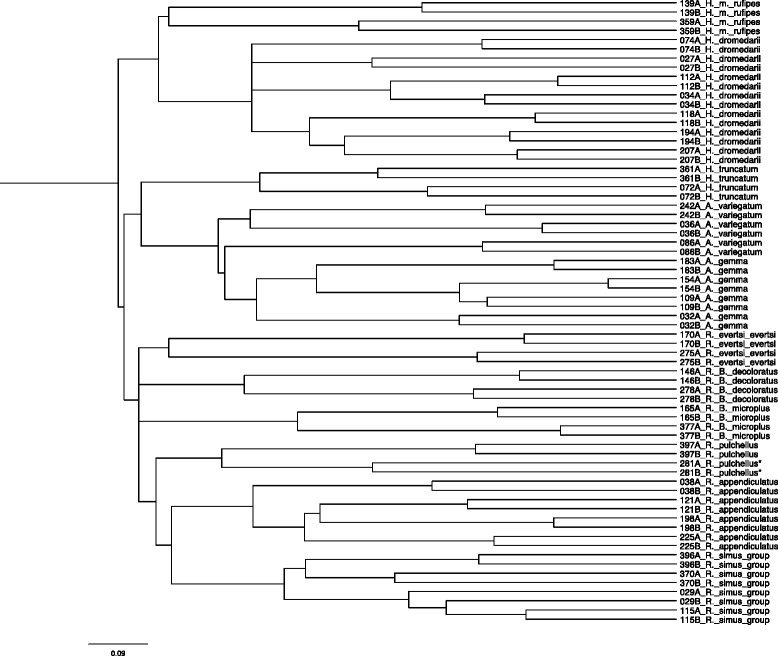
Cladogram (neighbour joining algorithm) illustrating the inter species specificity of tick mass spectra. Spectral profiles of two technical replicates (A and B) of the 33 COI gene sequenced ticks were integrated to the analysis. Two spectra (*) of a solely morphologically identified *R. pulchellus* tick were added to the dataset to maintain a minimum number of two specimens per species

To assess the interspecies specificity of the mass spectra generated by MALDI-TOF MS, the spectral profiles of the previous molecularly identified 33 ticks were subjected to cluster analysis (Fig. 3). As expected, the spectra derived from the same tick e.g. the technical replicates each clustered together in closest proximity. This was not the case for the mass profiles of just one tick (specimen no. 029). Importantly, within this set of ticks, all spectra derived from specimens of the same species seemed to share distinct masses that separated them clearly from the remaining tick species.

#### Definition of superspectra identifying East African tick species

After COI molecular and MALDI-TOF MS analysis, our specimen collection was slightly reduced from 398 to 381 ticks, now grouping into ten different species and the ambiguous *R. simus* group. Incorporating these results, SSp. were designed from a total of 48 ticks (Table 3). The mass profiles derived from the Kiambu laboratory tick strain that has been maintained for many years at the ILRI tick unit showed consistently high deviations from the other *R. appendiculatus* profiles (indicated in Fig. 3). This lead us to define a distinct SSp. designated as *R. appendiculatus* II exclusively covering this batch of ticks. The final 12 SSp. designed in this study were based on 192 mass spectra of 48 individual ticks and consisted of 14 to 30 individual protein masses (Table 3). In addition to including 24 COI typed specimens to the SSp. design, we also incorporated ticks representative of the diversity within a given species. This led to the inclusion of both field and laboratory ticks in some cases and to the inclusion of ticks with distinct mass patterns in other cases.

**Table 3:** Superspectra designed in this study

Name of SSp.	Condensed Mass Count	N (COI-typed)	Origin (N)
*Amblyomma gemma*	24	4 (2)	Field (4)
*Amblyomma variegatum*	30	4 (2)	Lab (3), Field (1)
*Hyalomma dromedarii*	29	4 (4)	Lab (4)
*Hyalomma marginatum rufipes*	24	4 (2)	Field (4)
*Hyalomma truncatum*	23	4 (2)	Field (4)
*Rhipicephalus (Boophilus) decoloratus*	14	4 (1)	Lab (2), Field (2)
*Rhipicephalus (Boophilus) microplus*	16	4 (2)	Lab (3), Field (1)
*Rhipicephalus appendiculatus* I	21	4 (3)	Lab Muguga (2), Field (2)
*Rhipicephalus appendiculatus* II	18	4 (1)	Lab Kiambu (4)
*Rhipicephalus evertsi evertsi*	19	4 (2)	Lab (2), Field (2)
*Rhipicephalus pulchellus*	26	4 (1)	Field (4)
*Rhipicephalus simus* group	18	4 (2)	Field (4)
Total		48 (24)	Lab (20), Field (28)

#### Validation of defined superspectra for tick identification

After removal of the 48 ticks used to build the reference spectra, 333 ticks remained for the validation step of the generated SSp. Our approach failed to assign an ID to 13 specimens, 319 ticks were correctly identified and only one tick was assigned a wrong ID. This corresponded to an overall sensitivity of 96.1 % and a specificity of 99.7 % (Table 4). Among the correctly identified ticks, 182 or 57.1 % matched with the correct SSp. in all four acquired mass spectra (indicated as 4× CC in Table 4). Sixty-six (20.7 %) ticks matched with three mass spectra to the correct SSp. while one mass spectrum resulted in no match. Twenty-three (7.2 %) ticks matched with two mass spectra to the correct SSp. while two mass spectra achieved no match. Nineteen (6.0 %) ticks matched with three mass spectra to the correct SSp. while one mass spectrum reached multiple matches, with the correct SSp. as the top match. The remaining 29 (9.1 %) ticks were positively identified, with their mass spectra matching in other combinations (indicated as “other” in Table 4).

**Table 4:** Validation of SSp. with 333 ticks

Tick species name	N	True ID assigned^a^					No ID assigned^a^	Wrong ID assigned^a^	Sensitivity	Specificity
		4× CC	3× CC 1× N	2× CC 2× N	3× CC 1× C	other				
*Amblyomma gemma*	21	4	4	5	0	4	4	0	81.00 %	100.00 %
*Amblyomma variegatum*	36	16	9	4	4	3	0	0	100.00 %	100.00 %
*Hyalomma dromedarii*	15	11	1	0	1	0	2	0	86.70 %	100.00 %
*Hyalomma marginatum rufipes*	14	4	4	2	0	2	2	0	85.70 %	100.00 %
*Hyalomma truncatum*	10	2	4	2	1	0	1	0	90.00 %	100.00 %
*Rhipicephalus (Boophilus) decoloratus*	37	25	7	1	2	2	0	0	100.00 %	100.00 %
*Rhipicephalus (Boophilus) microplus*	20	6	8	0	3	3	0	0	100.00 %	100.00 %
*Rhipicephalus appendiculatus*	74	53	13	3	2	2	1	0	98.60 %	100.00 %
*Rhipicephalus appendiculatus* (Kiambu)	5	4	0	0	0	0	1	0	80.00 %	100.00 %
*Rhipicephalus evertsi evertsi*	51	34	9	1	4	1	1	1	98.00 %	98.00 %
*Rhipicephalus pulchellus*	33	15	5	4	2	7	0	0	100.00 %	100.00 %
*Rhipicephalus simus* group	17	8	2	1	0	5	1	0	94.10 %	100.00 %
		182	66	23	19	29				
Total	333	319					13	1	96.10 %	99.70 %

^a^For each tick, four technical replicate mass spectra were matched against designed SSp. and a final ID assigned accordingly

CC: one correct SSp. matching; C: multiple SSp. matching, correct SSp. as top match; N: no matching SSp

other: true ID was assigned based on a different combination

**Fig. 4:**
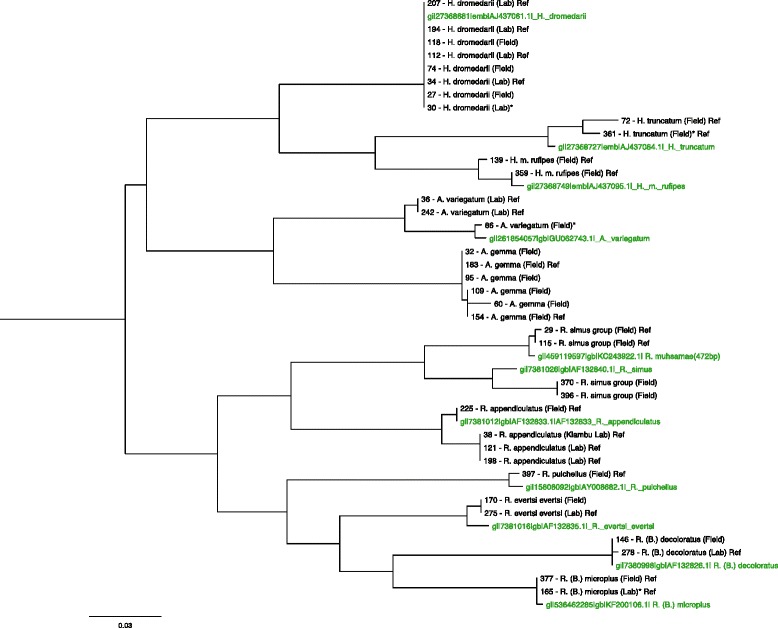
Phylogenetic relationship of 11 reference (NCBI) and 36 study ticks based on their COI gene sequences, illustrated as a maximum likelihood phylogenetic tree. Accentuated in green are top matching GenBank reference sequences. *Asterisks* (*) indicate non-consensus sequences. *Scale*: The bar length corresponds to 0.03 % (20 nt) difference in nucleotide sequence. *Ref*: Reference tick used to design SSp

Among the successfully identified ticks, the sensitivity of our approach was lowest for *A. gemma* (81 %) where a total of 21 ticks were used for validation and the *R. appendiculatus* ticks derived from the Kiambu stock (80 %), where only five ticks were used for validation. The sole tick that was assigned a false species ID, was a specimen morphologically identified as *R. evertsi evertsi.* While three of this tick’s mass spectra did not achieve a match at all, one mass spectrum was marginally similar (78 %, data not shown) to the *R. simus* group SSp. The mass spectra of the 13 ticks that could not be assigned to any SSp. and the spectrum of the wrongly assigned tick were inspected visually to assess the spectral quality. It appeared that most spectra displayed alterations like distorted or shifted mass peaks. The species identity of the wrongly identified *R. evertsi evertsi* specimen could not be verified on a molecular basis, since the extracted gDNA was not qualitatively sufficient for PCR. Three ticks with no SSp. ID (1× *H. dromedarii* and 2× *A. gemma*) were subjected to molecular COI analysis. The morphologically assigned species identities of all three specimens (Fig. 4; tick no. 95, 60 and 30) were confirmed on molecular basis.

The 48 ticks initially used to build the reference SSp. were not considered for the study validation. These mass spectra were later experimentally validated against all SSp. (data not shown). All mass spectra of the ticks were, as one would expect, correctly identified with their corresponding SSp.

## Discussion

Several genera of ixodid tick genera co-exist throughout Eastern Africa, including *Hyalomma*, *Amblyomma* and *Rhipicephalus*. Precise and timely data on tick population distribution and size in a given geographical area are required to model epidemiological trends of tick-borne diseases and formulate effective control strategies [1, 33]. However, tick identification by morphology can be limited by a lack of expertise, the need of several male specimens, whereas immature tick stages are difficult to identify by morphology alone [6].

In this study, MALDI-TOF MS was used to investigate a collection of laboratory-bred and field-collected afrotropical ixodid ticks with the aim of confirming their identity and establishing a reference MS spectra index designated as SSp.

The quality of the spectra generated for the vast majority of the ticks included in this study corresponded to what has been observed in similar studies with a range of arthropod vectors including European tick species [15, 16], tsetse flies [14], mosquitoes [12] and midges [31].

We found that spectra quality, overall protein mass counts and the molecular weight range that can be determined mainly depend on the initial quality of the sample itself. Seventeen ticks that were improperly stored and overgrown by fungus, or that were completely desiccated needed to be removed from this study due to inadequate quality of mass spectra obtained. A less apparent factor negatively affecting the overall spectral quality seems to be long-term storage of tick specimens in ethanol [31]. This could have been a factor in the failure to correctly identify 14 ticks, where most mass profiles revealed alterations in spectral quality on close examination. Poor peak resolution, diffuse signals in the low molecular weight range, and a shift in peak patterns were the most common characteristics observed in spectra from the unidentified specimens. Additional MALDI-TOF MS analysis with ticks plucked directly from animals or collected from vegetation without prior storage in ethanol could support these assumptions and reveal if spectral quality and taxonomic resolution can be enhanced significantly using freshly collected ticks.

In the majority of cases with samples sufficiently conserved, identification of ticks by matching their mass profiles against reference SSp. proved to be very robust. This was demonstrated by the high sensitivity (96.1 %) with which tick species were identified successfully. This is a significant achievement considering the large tick collection size, with some of the species represented by specimens originating from very different laboratory or field environments.

Together with COI gene sequences, a number of interesting conclusions can be inferred from the present study. The known problem of morphological tick species misidentification can be exemplified by two of our findings. (a) COI-typing of two ticks morphologically identified as *A. hebraeum*, revealed that the specimens were in fact members of the closely related *A. gemma*. This was for the most part resolved by our MALDI-TOF MS analyses, where three of the tick samples were identified clearly as *A. gemma*. The fourth tick, although showing high similarity with the *A. gemma* SSp., displayed spectral alterations and was not assigned any ID. (b) Similarly, a batch of ticks included into our collection clearly belonged to the genus *Hyalomma*. However, the absence of any reference specimen did not allow reliable morphological species identification. COI gene sequencing and MALDI-TOF MS both convincingly identified these ticks as *H. dromedarii*. These examples confirm the value of MALDI-TOF MS for resolution of tick taxonomic ambiguities. MALDI-TOF MS can provide improved and fast discrimination, especially when morphological examination is insufficient for a clear species designation.

The limitations of conventional tick typing are not restricted to the morphological approach but can extend to molecular techniques such as sequencing of the mitochondrial COI gene. This issue has been highlighted by the example of genetic hybridization occurring amongst members of the genus *Hyalomma* as described by Rees et al. [34]. While individuals of the species *H. truncatum*, *H. dromedarii* and *H. marginatum rufipes* are well differentiated both morphologically and genetically, sexual reproduction between members of these species can occur, resulting in hybrid offspring. Such intermediate individuals (e.g. NCBI record AJ437088.1 in Table 2) still display the distinct paternal morphological features while possessing the maternally inherited mtDNA genotype. The use of COI sequencing on its own can therefore result in misclassification of such specimens. It will be the subject of further research to establish how the mass spectra of hybrid ticks differ from the parental protein fingerprint and to what extent MALDI-TOF MS can serve as monitoring tool for following the gene flow amongst different tick species.

One unresolved issue in African tick taxonomy was highlighted by our findings regarding the ticks morphologically assigned as *R. praetextatus* and *R. simus*. The ongoing debate, as to which of these species is distributed where in East-Africa is based largely on the fact that they can not be easily separated morphologically [5]. Defining the accurate spread of *R. simus* and *R. praetextatus* is further complicated by the co-occurrence of other, highly similar species including *R. lunulatus* and *R. muhsamae* known to be present in the same East African habitats [5, 35]. The fact, that *R. simus* has been described to be restricted to Southern Africa [4, 5] suggested early on that our *R. simus* field isolates from Kenya were mistaken with a morphologically highly similar species. This was supported by the COI gene analysis, which grouped this ticks closer to *R. praetextatus* (BOLD, 94 % identity) and *R. muhsamae* (GenBank, 99 % identity and 67 % query cover) than to *R. simus* (GenBank, 92 % identity and 99 % query cover). The sequence data was equally unclear for our *R. praetextatus* specimens, with both high matches to unpublished records of *R. praetextatus* reference sequences on BOLD (100 % identity) and *R. simus* in GenBank (96 % identity, 98 % query cover). A phylogenetic maximum-likelihood analysis of all COI nucleotide sequences derived in this study and reference records from GenBank (Fig. 4), supports these inferences regarding *R. simus* and *R. praetextatus*. Although there appears to be a clear molecular boundary between the analyzed ticks with a suggested close proximity of the *R. simus* ticks (specimen no. 29 and 115) to *R. muhsamae*, the limited reference records available do not allow a conclusive answer regarding the true identity of our ticks. We therefore merged these ticks to one group defined as *R. simus* group, enveloping the tick species *R. simus*, *R. praetextatus* and *R. muhsamae*, as previously suggested by Walker, Keirans and Horak [5].

Whether MALDI-TOF MS analysis can distinguish between these three tick species, where current COI, 12S and ITS2 molecular data is non-conclusive (E. Kanduma and R. Bishop, unpublished data), requires further investigation with representative specimens from all three species. It is however worth noting that our phylogenetic cluster analysis of four specimens designated as members of the *R. simus* group indicated potential differences between the spectral profiles (Fig. 3). Further studies will be needed to conclusively confirm the value of MALDI-TOF MS in discriminating between the species of the *R. simus* group.

The Kiambu *R. appendiculatus* specimens, where only five ticks were available for validation, and the ticks belonging to *A. gemma* were detected with the lowest sensitivity by our SSp. approach. The failure in species ID assignment for these specimens might partially be explained by the negative effect of long-term storage in ethanol as discussed before. Additionally, we hypothesized that intraspecies genetic heterogeneity could be increased in these two sets of ticks, leading to stronger diversity in spectral fingerprints. Phylogenetic analysis of the COI gene sequences does not support this theory (Fig. 4). The *A. gemma* ticks among each other, as well as the *R. appendiculatus* ticks of both Muguga and Kiambu stock shared almost identical COI nucleotide sequences. Continuative studies, incorporating freshly plucked ticks, will help to determine to what extent the overall sensitivity of a SSp. based identification approach can still be improved.

Looking ahead, the potential applications of MALDI-TOF MS as a tick species typing tool are diverse, ranging from pathogen and vector epidemiological monitoring for disease outbreak detection, to following consequences of climate change and its influence on changing patterns of tick distribution and its associated disease risks as described [36]. Furthermore, the SSp. established here provide the basis to move towards simultaneous characterization of African tick vectors and pathogens transmitted by MALDI-TOF MS, as has been shown with *Rickettsia* [37]. Another immediate use of this technique would be monitoring the spread of the invasive single host tick *R*. (*B.*) *microplus* [38]. This tick is both more adaptable to changing environments than native species like *R.* (*B.*) *decoloratus* and has greater potency in transmission of protozoan and bacterial pathogens, including *Babesia bigemina, Babesia bovis* and *Anaplasma marginale* [39].

## Conclusions

In summary, our study demonstrated the applicability of MALDI-TOF MS as a suitable tool for East African tick identification. The processing steps of the ticks for MALDI-TOF MS are straightforward, with little time and human and equipment resources needed. The rapid generation of mass spectra profiles and their automated, immediate comparison against pre-designed reference SSp. allow high-throughput measurement of large numbers of samples. We identified the quality of the samples used as the main limiting factor for the MALDI-TOF MS analyses. Whenever possible, tick material collected freshly from the field should be analyzed. The negative impact of sample storage under ethanol for limited periods of time should be evaluated carefully, since this would increase applicability to large tick collections sampled across Africa. Under good conditions of sample storage, MALDI-TOF MS can generate highly distinctive mass profile patterns that will allow precise and rapid monitoring of tick populations, species movements, pathogen transmission and host feeding preferences on a large-scale.

## Additional file

Additional file 1: Figure S1. Comparison of MALDI-TOF MS spectral profiles, indicating distinct mass peak patterns among the different tick genera *Amblyomma*, *Hyalomma, Rhipicephalus* and *Rhipicephalus* (*Boophilus*). The spectra illustrated in the figure cover a mass range between 4000 to 18,000 Da. The relative peak intensities are indicated on the y-axis. (PDF 143 kb)

## Abbreviations

BOLD: Barcode of Life Database
bp: base pair
COI: cytochrome *c* oxidase I
DNA: deoxyribonucleic acid
ILRI: International Livestock Research Institute
NCBI: National Center Bioinformatics
nt: nucleotide
MALDI: matrix-assisted laser desorption/ionization
MS: mass spectrometry
PCR: polymerase chain reaction
ppm: parts per million
SSp.: superspectrum
sp.: species
TOF: time-of-flight
