# Supplementary figures and images for: Matrix-assisted laser desorption/ionization time of flight mass spectrometry for comprehensive indexing of East African ixodid tick species
Source: Parasit Vectors. 2016 Mar 15;9:151. doi: 10.1186/s13071-016-1424-6 (PMC4792108; doi:10.1186/s13071-016-1424-6)

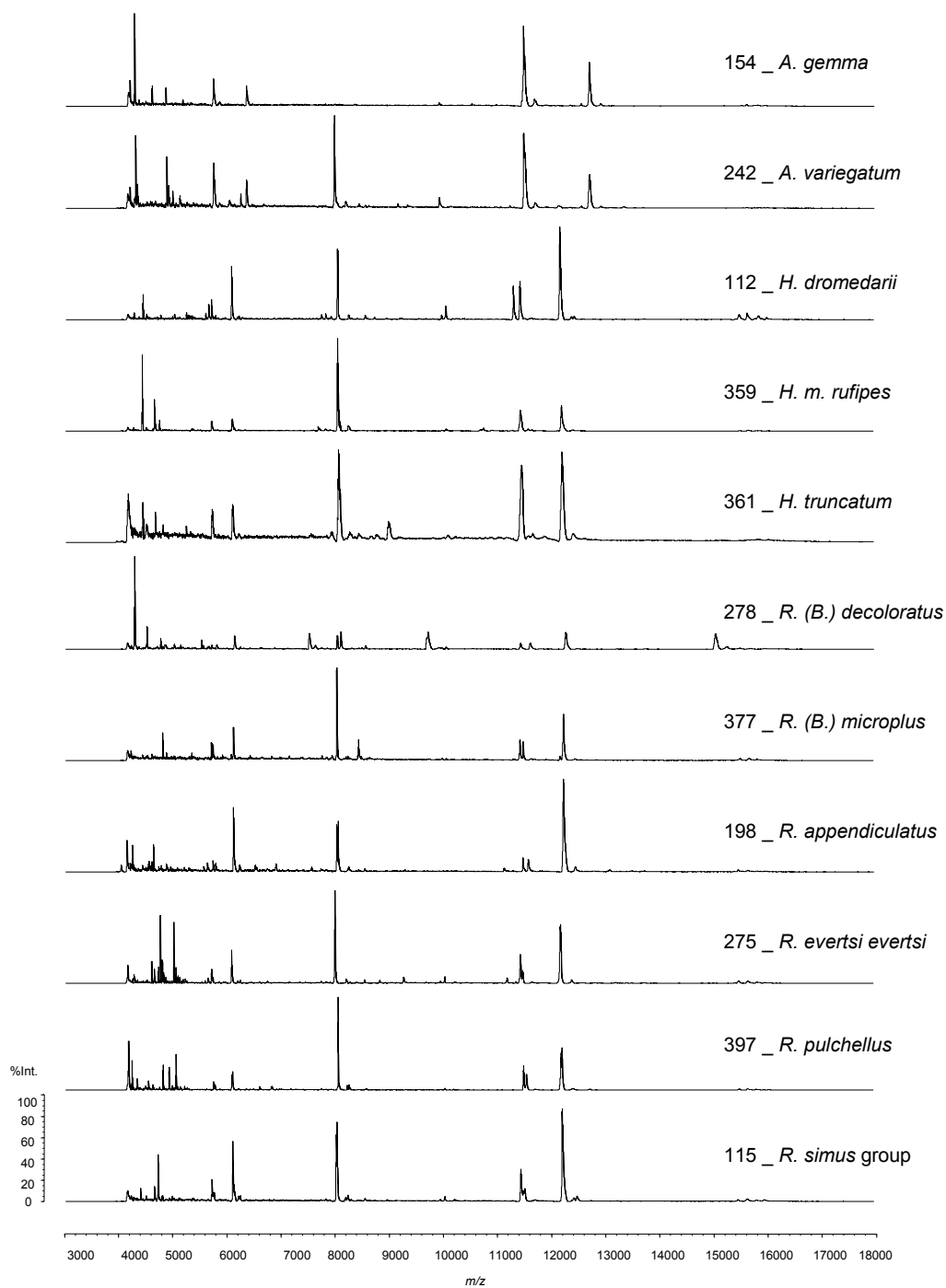

Supplement: Additional file 1: Figure S1. — Comparison of MALDI-TOF MS spectral profiles, indicating distinct mass peak patterns among the different tick genera Amblyomma, Hyalomma, Rhipicephalus and Rhipicephalus (Boophilus). The spectra illustrated in the figure cover a mass range between 4000 to 18,000 Da. The relative peak intensities are indicated on the y-axis. (PDF 143 kb) [file 13071_2016_1424_MOESM1_ESM.pdf]
